# The *FCGR2C* allele that modulated the risk of HIV-1 infection in the Thai RV144 vaccine trial is implicated in HIV-1 disease progression

**DOI:** 10.1038/s41435-018-0053-9

**Published:** 2018-12-19

**Authors:** Ria Lassaunière, Maria Paximadis, Osman Ebrahim, Richard E. Chaisson, Neil A. Martinson, Caroline T. Tiemessen

**Affiliations:** 1 National Institute for Communicable Diseases, Centre for HIV and STI’s, Johannesburg, South Africa; 2 University of the Witwatersrand, Faculty of Health Sciences, Johannesburg, South Africa; 3 Department of Virus and Microbiological Special Diagnostics, Statens Serum Institut, Copenhagen, Denmark; 4 Brenthurst Clinic, Johannesburg, South Africa; 5 Johns Hopkins University Center for AIDS Research, Baltimore, MD USA; 6 Perinatal HIV Research Unit (PHRU), University of the Witwatersrand, Johannesburg, South Africa; 7 MRC Soweto Matlosana Centre for HIV/AIDS and TB Research, Johannesburg, South Africa

**Keywords:** Disease genetics, Infection

## Abstract

In the HIV-1 Thai RV144 vaccine trial—the only trial to demonstrate any vaccine efficacy to date—a three-variant haplotype within the Fc gamma receptor 2C gene (*FCGR2C*) modified the risk of HIV-1 acquisition. A similar vaccine regimen is currently being evaluated in South Africa in the HVTN702 trial, where the predominant population is polymorphic for only a single variant in the haplotype, c.134-96C>T. To investigate the significance of c.134-96C>T in HIV-specific immunity in South Africans, this study assessed its role in HIV-1 disease progression. In a cohort of HIV-1-infected South African controllers (*n* = 71) and progressors (*n* = 73), the c.134-96C>T minor allele significantly associated with increased odds of HIV-1 disease progression (odds ratio 3.80, 95% confidence interval 1.90–7.62; *P* = 2.0 × 10^–4^, *P*_Bonf_ = 2.4 × 10^–3^). It is unlikely that the underlying mechanism involves wild-type FcγRIIc function, since only a single study participant was predicted to express wild-type FcγRIIc as determined by the *FCGR2C* c.798+1A>G splice-site variant. Conversely, in silico analysis revealed a potential role for c.134-96C> T in modulating mRNA transcription. In conclusion, these data provide additional evidence towards a role for *FCGR2C* c.134-96C>T in the context of HIV-1 and underscore the need to investigate its significance in the HVTN702 efficacy trial in South Africa.

## Introduction

Immune effector functions recruited through the Fc portion of immunoglobulin G (IgG) are increasingly recognised as an important component of HIV-1 protective immunity [1]. In murine and non-human primate models, Fc-mediated mechanisms have been demonstrated to augment the in vivo activity of broadly neutralizing antibodies [2, 3], whereas robust antibody-dependent cellular cytotoxicity (ADCC) responses in humans have been associated with reduced risk of HIV-1 infection following immunization [4], reduced perinatal HIV-1 transmission risk [5] and slower disease progression [6, 7].

IgG Fc-mediated effector functions are predominantly recruited through the engagement of Fc gamma receptors (FcγRs)—a family of polymorphic glycoproteins encoded by five genes, *FCGR2A/B/C* and *FCGR3A/B*. In the phase 3 Thai RV144 vaccine trial, which evaluated a prime-boost vaccination regimen of ALVAC-HIV (vCP1521) and HIV-1 gp120 AIDSVAX B/E, a novel *FCGR2C* haplotype significantly modified vaccine efficacy [8, 9]. This haplotype—hereafter referred to as the Thai *FCGR2C* haplotype—comprised three single-nucleotide variants that were in complete linkage disequilibrium, including c.353C>T (p.T118I, rs138747765) in *FCGR2C* exon 3, c.134-96C>T (rs114945036) upstream of exon 3 and c.391+111G>A (rs78603008) downstream of exon 3. Thai vaccinees who possessed at least one minor allele of the c.134-96C>T tag variant, had an estimated vaccine efficacy of 91% against CRF01_AE 169K HIV-1 and 64% against any HIV-1 strain, compared to 15 and 11% in vaccinees homozygous for the wild-type allele, respectively [8].

Building on the successes of the RV144 trial, a similar prime-boost vaccine regimen—ALVAC-HIV (vCP2438)+bivalent subtype C gp120/MF59—is currently being evaluated in the HVTN702 phase 2b efficacy trial in South Africa. The Thai and South African populations are, however, distinctly different at the *FCGR2C* gene locus. The three variants in the Thai*FCGR2C* haplotype are not in complete linkage disequilibrium in the predominant black South African population. Only the c.134-96C>T variant has an appreciable minor allele frequency of 24.9%, whereas the other two variants—c.391+111G>A and p.T118I—rarely occur [10]. It is unclear if the population differences will differentially affect HIV-1 vaccine protection in Thais and South Africans, since the causal variant associated with the Thai *FCGR2C* haplotype is unknown and the significance of the c.134-96C>T variant in Africans has not been studied. Here we investigated the c.134-96C>T variant and other functional *FCGR2C* variants in the context of HIV-1 immunity in black South Africans by determining their association with HIV-1 disease progression.

## Results

### Study design and subjects

A case–control candidate gene association study was undertaken to assess the significance of *FCGR2C* variants in HIV-1 disease progression. Here we employed selective genotyping, whereby HIV-1-infected individuals at the extreme ends of the HIV-1 disease progression phenotype, that is HIV-1 controllers and chronic progressors, were selected and their *FCGR2C* genotypes were compared. This approach, also called ‘extreme phenotype sampling’, has increased statistical power over random sampling of the same sample size and is effective for detecting genetic effects in complex disease traits [11, 12].

HIV-1 progressors (*n* = 73) were selected from the Soweto Lung Cohort (*n* = 756) recruited at Chris Hani Baragwaneth hospital using the following criteria: (i) CD4 T-cell decline from >500 cells/μl to <350 cells/μl; (ii) initiated antiretroviral therapy and (iii) >10,000 HIV-1 RNA copies/ml at the time of antiretroviral therapy initiation. The HIV-1 controller phenotypes were defined as follows: elite controllers (*n* = 23) had at least one HIV RNA determination of <50 copies/ml and CD4 T-cell counts >500 cells/μl; viraemic controllers (*n* = 37) had viral load set points between 50 and 2000 copies/ml and CD4 T-cell counts >500 cells/μl and high viral load long-term non-progressors (*n* = 11) had multiple viral load measurements >10,000 RNA copies/ml and CD4 T-cell counts >500 cells/μl without apparent CD4 T-cell decline for a period of ≥7 years.

While the three HIV-1 controller groups differ in their ability to control viraemia, all maintained comparatively high CD4 T-cell counts that were significantly higher compared to the chronic progressor group (*P* < 0.0001 for all comparisons, Table 1). Overall, the study participants were predominantly female (84.7%) with a median age of 38 years (interquartile range [IQR] 33–42.5 years). Gender and age did not differ significantly between the HIV-1 controllers (total and individual controller groups) and HIV-1 progressors (*P* > 0.05 for all comparisons).

**Table 1:** Clinical and demographic characteristics of HIV-1 disease phenotype groups

	Elite controllers	Viraemic controllers	High viral load long-term non-progressors	Chronic progressors
*N*	23	37	11	73
*Age (years) at enrolment*	40.7 (9.7)	35.4 (8.7)	40.3 (6.9)	38.4 (7.3)
*Gender* % Females	78.3	91.9	81.8	83.6
*CD4 T-cell count (cells/µl)*^**a**^	784 (371)	735 (233)	682 (109)	173 (63)
*Viral load (copies/ml)*^**a**^	< 20	598 (237–1270)	22410 (11,370–81,325)	39,322 (19,822–105,195)
*Time since diagnosis (years)*	10 (4–12)	3 (2–11)	8 (8–11)	6 (1–7)

Mean with standard deviation is reported for 'Age' and 'CD4 T cell count'; median with interquartile range is reported for 'Viral load' and 'Time since diagnosis'. For HIV-1 controllers the CD4 T cell count and viral load at study enrolment was used, whereas for HIV-1 chronic progressors the CD4 T cell count and viral load prior to initiation of antiretroviral therapy was used

### *FCGR2C* copy number does not associate with HIV-1 disease progression

**Fig. 1:**
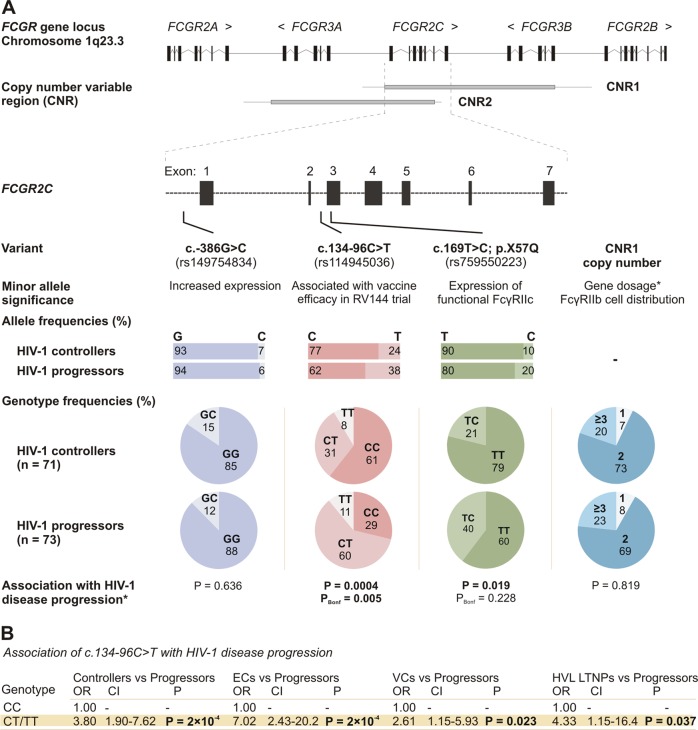
Functional and clinically significant *FCGR2C* variants and their genotype distribution in HIV-1-infected South Africans with differential control of HIV-1 infection—controllers (*n* = 71) and progressors (*n* = 73). **a***FCGR* arrangement on chromosome 1q23.3. *FCGR2C* is duplicated/deleted in two different genomic segments, copy number variable region 1 (CNR1) and CNR2 (grey bars). *FCGR2C* variants with a minor allele frequency above 5%—c.-386G>C, c.134-96C>T, c.169T>C (p.X57Q) and CNR1—were assessed for an association with HIV-1 disease progression. **b** Odds ratios (OR), confidence intervals (CI) and *P*-values for the c.134-96C>T association in different HIV-1 controller groups—elite controllers (ECs), viraemic controllers (VCs), and high viral load long-term non-progressors (HVL LTNPs). *Fisher’s exact test of genotype distributions

The low-affinity *FCGR* locus on chromosome 1q23 is subject to copy number variation, with genes duplicated or deleted within distinct genomic copy number variable regions (CNRs). In this study cohort, the gain or loss of an *FCGR2C* gene copy was observed only within the previously designated CNR1 or CNR2 (Fig. 1), where CNR1 encompasses a complete copy of *FCGR2C* and CNR2 an incomplete copy (lacks the last exon). The two CNRs further differ with regard to the syntenic genes that are duplicated/deleted as well as the associated phenotypic and/or functional consequences. The variability of CNR1 and CNR2 was therefore assessed independently.

Similar to healthy HIV-uninfected South Africans and other population groups, copy number variability of CNR1 was observed more frequently than CNR2 in the 144 HIV-1 infected study participants (29.2 vs. 4.2%). The copy number distribution of CNR1 was comparable between HIV-1 controllers (total and phenotype groups) and HIV-1 progressors (*P* > 0.05 for all comparisons) (Fig. 1), whereas an association between CNR2 and HIV-1 disease progression was not assessed due to the low frequency of CNR2 variability (<5%).

### *FCGR2C* c.134-96C>T (rs114945036) associates with increased odds of HIV-1 disease progression

Study participants were genotyped for the following functionally and/or clinically significant *FCGR2C* nucleotide variants: (i) c.–386G>C (rs149754834) that modulates gene expression levels; (ii) c.134-96C>T (rs114945036) that associated with risk of HIV-1 acquisition in the RV144 HIV-1 vaccine trial; (iii) c.169T>C (p.X57Q) (rs759550223) that alters the open-reading frame and predicts FcγRIIc expression together with (iv) c.798+1A>G (rs76277413) that modifies pre-mRNA splicing. The latter had a minor allele frequency of <0.1% and was thus not included in further analysis. Variable *FCGR2C* copy number individuals were included in subsequent analysis as follows: (i) individuals with more than two *FCGR2C* copies were considered homozygous when all gene copies carried the same allele and heterozygous when both alleles were present; (ii) individuals bearing a single*FCGR2C* copy were considered homozygous. Associations of *FCGR2C* variants were unadjusted for *FCGR2C* copy number since the latter did not independently associate with HIV-1 disease progression.

The genotype distribution of c.134-96C>T was significantly different between HIV-1 controllers and progressors (*P* = 4.2 × 10^–4^, *P*_Bonf_ = 0.005; Fig. 1). In particular, the c.134-96T allele was overrepresented in HIV-1 progressors compared to HIV-1 controllers (37.7% vs. 23.5%) and significantly associated with increased odds of HIV-1 disease progression in a dominant model (odds ratio [OR] 3.80, 95% confidence interval [CI] 1.90–7.62; *P* = 2.0 × 10^–4^, *P*_Bonf_ = 2.4 × 10^–3^). This association was the strongest for the elite controller group (OR 7.02, 95% CI 2.43–20.25; *P* = 1.8 × 10^–4^; *P*_Bonf_ = 2.2 × 10^–3^) compared to the viraemic controller group (OR 2.61, 95% CI 1.15–5.93; *P* = 0.023, *P*_Bonf_ > 0.05) or high viral load LTNP group (OR 4.33, 95% CI 1.14–16.37; *P* = 0.037,*P*_Bonf_ > 0.05) (Fig. 1).

A weaker association was also observed for the c.169T>C (p.X57Q) variant (*P* = 0.019, *P*_Bonf_ > 0.05; Fig. 1). The c.169C allele, which maintains the open-reading frame and is required for expression of functional FcγRIIc, was overrepresented in HIV-1 progressors compared to HIV-1 controllers (19.8% vs. 10.1%) and associated with increased odds of HIV-1 disease progression in a dominant model [OR 2.46, 95% CI 1.18–5.14; *P* = 0.017, *P*_Bonf_ > 0.05). The association was not significant when HIV-1 controller phenotypes were assessed independently (*P* > 0.05 for all comparisons). Notably, whilst 44/144 (30.6%) study participants carried a c.169C allele, only one expressed membrane-bound FcγRIIc as predicted by the c.798+1A>G splice-site variant. Thus, the observed association between the c.169C allele and HIV-1 disease progression is likely unrelated to expression of membrane-bound FcγRIIc.

### Strong linkage disequilibrium between *FCGR2C* c.134-96C>T and c.169T>C

**Fig. 2:**
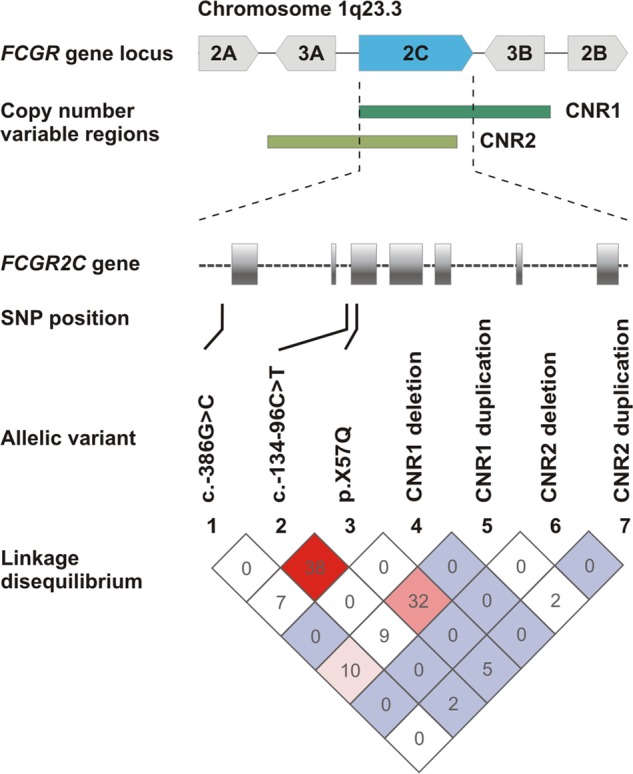
Linkage disquilibrium of *FCGR2C* genotypic variants—gene copy number and single-nucleotide polymorphisms—in HIV-1-infected South Africans. CNR1 and CNR2—copy number variable regions 1 and 2, genomic segments in which a *FCGR2C* copy was duplicated or deleted. Values and colours reflect *r*^2^ (×100) and*D*′/LOD measures of LD, respectively

To determine if the associations observed for c.134-96C>T and c.169T>C are linked due to co-inheritance of minor alleles, we next assessed the linkage disequilibrium between the different *FCGR2C* variants (Fig. 2). Indeed, strong linkage disequilibrium existed between c.134-96C>T and c.169 T > C, both when unadjusted for copy number variability (*D*′ = 1 and *r*^2^ = 0.382, *P* < 0.001) and when individuals with only two*FCGR2C* copies were considered (*D*′ = 1 and *r*^2^ = 0.227, *P* < 0.001). In particular, the less frequent c.169C (p.57Q) allele always occurred in the presence of the more frequent c.134-96T allele. Following adjustment for c.169T>C in a multivariate logistic regression model, the association between c.134-96C>T and HIV-1 disease progression remained significant (OR 3.62, 95% CI 1.54–8.54; *P* = 0.003, *P*_Bonf_ = 0.036).

### *FCGR2C* c.134-96C>T is in strong linkage disequilibrium with other *FCGR2C* variants in native Africans

The other constituents in the Thai *FCGR2C* haplotype, p.T118I (rs138747765) and c.391+111G>A (rs78603008), have minor allele frequencies of <1% in native African populations [10]. Where detected in Africans, the minor alleles occur in complete linkage disequilibrium. Therefore, p.T118I was used as a tag variant for c.391+111G>A in the present study. A comparably low prevalence of the p.TII8I minor allele was observed in the HIV-1-infected cohort (0.7%), which suggests that the observed association of the c.134-96C>T variant with HIV-1 disease progression in South Africans was independent of the other Thai *FCGR2C* haplotype variants.

**Fig. 3:**
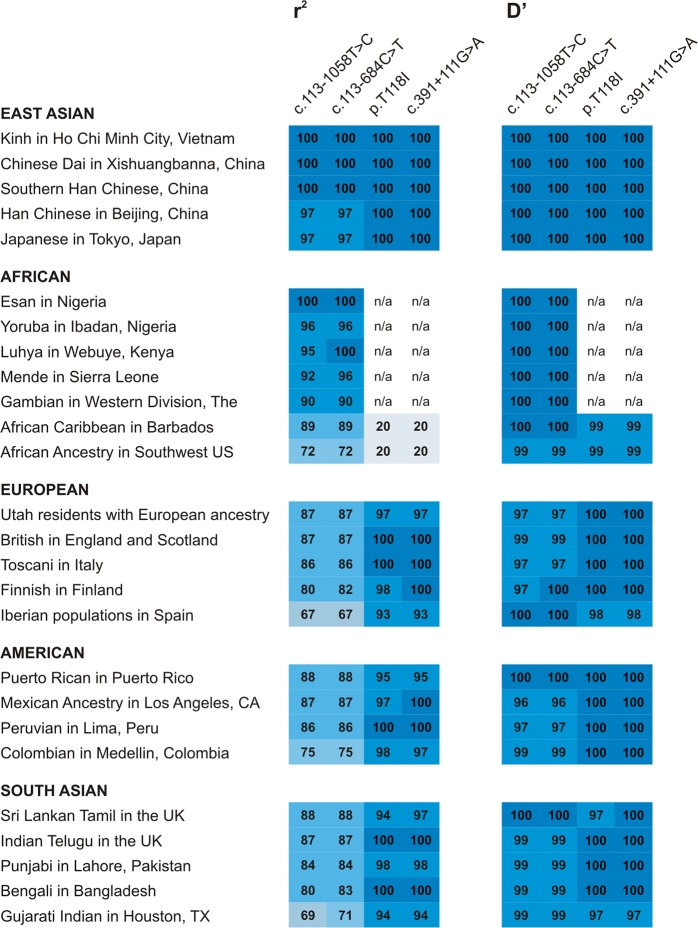
Global patterns of linkage disequilibrium between*FCGR2C* variants identified to be in strong-to-complete linkage disequilibrium with c.134-96C>T in this study (c.113-1058 T > C and c.113-684C>T) and in Thai vaccinees (p.T118I and c.391+111G>A). Population data were obtained from the 1000 Genomes Project. Values indicate *r*^2^ × 100 and*D′* × 100; n/a: not applicable, minor allele frequency too low

Full *FCGR2C* sequences of the South African study cohort were not available to enable identification of additional variants in linkage disequilibrium with c.134-96C>T. Thus,*FCGR2C* genotypic data from African populations in the 1000 Genomes Project were assessed. In native Africans and other population groups, two variants within *FCGR2C* intron 1—c.113-1058T>C (rs2169052/rs115953596) and c.113-684C>T (rs111828362)—were in strong-to-complete linkage disequilibrium with c.134-96C>T (*D′* ≥ 0.960 and *r*^2^ ≥ 0.900; and*D′* ≥ 0.960 and *r*^2^ ≥ 0.670, respectively) (Fig. 3). Notably, complete linkage disequilibrium (*D′* = 1 and *r*^2^ = 1) was observed between c.134-96C>T, c.113-1058T>C, c.113-684C>T, p.T118I and c.391+111G>A in two mainland Southeast Asia populations (Kinh in Vietnam and Chinese Dai in China) that share common ancestry with Thai populations [13]. These additional linkage patterns may be of significance for elucidating the mechanisms underlying the association of c.134-96C>T with vaccine efficacy in the Thai RV144 vaccine trial and HIV-1 disease progression; however, it requires confirmation with*FCGR2C* gene-specific approaches that account for gene copy number variability.

### In silico analysis of c.134-96C>T and linked variants

**Fig. 4:**
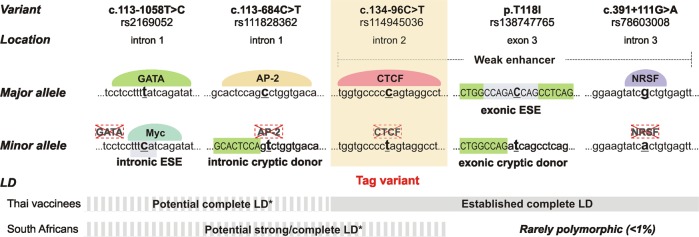
In silico-predicted implications of *FCGR2C* c.134-96C>T and linked variants on transcriptional regulatory motifs and splicing. Chromatin marks place the Thai *FCGR2C* haplotype—c.134-96C>T, p.T118I, c.391+111G>A—in a weak enhancer site, where the variants flanking exon 3 were predicted to disrupt binding of transcription factors CTCF and NRSF (presence/absence of coloured domes above the sequences). The p.T118I variant was predicted to disrupt an exonic splice enhancer (ESE) and create an exonic cryptic donor site, whereas the intron 1 variants c.113-1058T>C and c.113-684C>T were predicted to create an intronic ESE and intronic cryptic donor site, respectively. Bold underlined letters—nucleotide variant; capital letters shaded in green—exon sequences; small letters—intron sequences; nucleotide sequences shaded grey—ESEs; LD—linkage disequilibrium. *Inferred from the 1000 Genomes Project data for geographically close populations

To explore the potential mechanism(s) underlying the associations of c.134-96C>T with HIV-1 acquisition and disease progression, we studied in silico the potential impact of c.134-96C>T and linked variants on transcriptional regulation and splicing (summarized in Fig. 4).

Chromatin profiling locates the three variants in the Thai*FCGR2C* haplotype—c.134-96C>T, p.T118I, c.391+111G>A—in a weak transcriptional enhancer in seven tissues, i.e. embryonic stem cells, induced pluripotent stem cells, embryonic stem cell-derived cells, mesenchymal cells, epithelial cells, adrenal cells and the K562 leukaemia cell line. Within this regulatory element, c.134-96C>T was predicted to alter the putative binding motif for CCCTC-binding factor (CTCF) and c.391+111G>A, the binding motif for the transcriptional repressor neuron-restrictive silencer factor (NRSF). The intron 1 variants c.113-1058T>C and c.113-684C>T were not predicted to occur in transcriptional regulatory elements; however, they occur within putative regulatory protein-binding motifs for GATA and activator protein-2 (AP-2), respectively.

While none of the variants are located at intron/exon boundaries and, thus, do not affect splice sites directly, they may activate cryptic splice sites or disrupt/create auxiliary sequences that promote or repress splicing, i.e. enhancers and silencers. In silico analysis predicted that the minor allele of p.T118I activates a cryptic donor site and disrupts an exonic splice enhancer—a motif that directs accurate splicing by assisting in the recruitment of splicing factors to the adjacent intron—and alters the putative binding sites for serine and arginine-rich splicing factor 1 (SRSF1 or ASF1/SF2) and 5 (SRSF5 or SRp40). The intronic variants flanking exon 3 were not predicted to activate cryptic splice sites or modulate auxiliary sequences. However, the variants located in intron 1, c.113-684C>T and c.113-1058T>C, potentially activate a cryptic donor site and exonic sequence enhancer, respectively.

## Discussion

Accumulating evidence suggests that *FCGR2C* genetic variability is of clinical significance. The minor allele of *FCGR2C* c.169T>C (p.X57Q), which predicts expression of functional FcγRIIc, has been associated with autoimmune diseases [14, 15], HIV-associated tuberculosis [16] and antibody responses following vaccination [15]. Moreover, in the Thai phase 3 RV144 HIV-1 vaccine trial, an intragenic *FCGR2C* haplotype with c.134-96C>T (rs114945036) as tag variant, associated with reduced risk of HIV-1 infection following vaccination [8].

Here we describe an additional association between the *FCGR2C* c.134-96C>T tag variant and disease progression in HIV-1-infected South Africans. A weaker association was also observed for the c.169T>C (p.X57Q) variant. However, this may be the result of strong linkage disequilibrium with c.134-96C>T (*D′* = 1). The c.169T>C (p.X57Q) variant’s functional significance is also unclear since only a single study participant was predicted to express FcγRIIc based on the donor splice-site variant in intron 6 (c.789–1A>G) [10, 17]. An independent association for c.169T>C with HIV-1 disease progression cannot be excluded. However, in this study, the c.169T>C minor allele was not detected in the absence of the c.134-96C>T minor allele, which precluded such an analysis. Conversely, an independent association for the more prevalent c.134-96C>T variant was confirmed in a multivariate logistic regression model.

The association between the c.134-96C>T variant and HIV-1 disease progression was primarily determined by the elite controller group; however, weaker associations were also observed for the viraemic controller and high viral load long-term non-progressor groups. Since the controller groups had distinctly different viral loads, it is unclear if the c.134-96C>T variant plays a role in natural control of HIV-1 viraemia. Conversely, the three HIV-1 controller groups had comparatively high CD4 T-cell counts, all of which were significantly higher compared to the HIV-1 progressor group. We hypothesize therefore that the c.134-96C>T variant plays a role in modifying CD4 T-cell integrity in HIV-1-infected individuals.

The contrasting roles of the c.134-96C>T variant in protection against HIV-1 acquisition in Thai vaccinees in the RV144 trial and disease progression in HIV-1-infected South Africans are intriguing [8]. The functional significance of either association is currently unknown. It therefore remains to be established if a common mechanism, which is advantageous in protection from infection but deleterious once infected, is shared or if different mechanisms are involved in the two scenarios.

Potential mechanisms may involve modulation of gene expression, as shown in Epstein–Barr virus transformed lymphoblastoid B-cell lines from European Caucasians, where all variants within the Thai *FCGR2C* haplotype associated with increased expression levels of*FCGR2A* and/or *FCGR2C* exon 7 [18]. This observation may be explained by the occurrence of the Thai *FCGR2C* haplotype within a predicted weak transcriptional enhancer, where the minor alleles of c.134-96C>T and c.391+111G>A potentially alter putative binding motifs for the transcriptional repressor proteins CTCF and NRSF, respectively [19, 20]. If indeed the promoter of either *FCGR2A* or *FCGR2C* interacts with this enhancer element, abolished binding of the aforementioned DNA-binding proteins may increase mRNA expression as observed in immortalized B cells. However, an interaction between the enhancer and the promoters of *FCGR2A* and *FCGR2C* remains to be determined empirically. Additional investigations in this regard should consider the tissue specificity of enhancers and the effect of Epstein–Barr virus proteins on their function.

Li et al. proposed that variants within the Thai *FCGR2C* haplotype can potentially alter pre-mRNA splicing that may result in exon skipping [8]. Through in silico prediction, we demonstrate that p.T118I in exon 3 may disrupt an exonic splice enhancer, a known cause of exon skipping [21]. The two variants in *FCGR2C* intron 1 that are in strong linkage disequilibrium with c.134-96C>T in all 1000 Genomes Project populations were also predicted to create splice regulatory motifs. Future studies of mRNA transcripts from individuals with differential carriage of c.134-96C>T and linked variant alleles will be required to establish a role for altered splicing.

Consideration of population differences when studying the functional significance of c.134-96C>T is paramount. In Thai vaccinees, this variant was in complete linkage disequilibrium with p.T118I and c.391+111G>A [8], whereas in South Africans, the latter loci are rarely polymorphic [10]. Consequently, there may be different possibilities regarding causality. One is that p.T118I and c.391+111G>A within the haplotype bear little or no functional significance and that c.134-96C>T is causal or in linkage with a functional variant elsewhere, potentially c.113-684C>T and c.113-1058T>C in *FCGR2C* intron 1. Alternatively, the association observed in Thai vaccinees involves p.T118I and c.391+111G>A and consequently a separate mechanism. Nevertheless, both studies indicate the relevance for c.134-96C>T in the context of HIV-1 and warrant further investigation.

It should be noted that factors modulating the risk of HIV-1 acquisition following immunization and HIV-1 disease progression once infection is established may not necessarily overlap. For example, in Thai vaccinees, the HLA class I A*02 allele associated with protection from acquisition of HIV-1 CRF01_AE in the RV144 trial [22], but the same allele did not affect progression to disease in Thai HIV-1 CRF01_AE infected military recruits [23]. The findings of the present study are therefore not necessarily indicative of a deleterious role for c.134-96C>T in the HVTN702 vaccine trial. However, the data suggest that South African HVTN702 trial participants who acquire HIV-1 in the placebo arm may be at increased risk of disease progression if they carry the *FCGR2C* c.134-96C>T minor allele and are not treated. This increased risk may also occur for breakthrough infections in the vaccine arm should the vaccine have no impact on HIV-1 disease progression. Long-term follow-up of Thai RV144 trial participants with breakthrough infections will potentially elucidate the role of the complete Thai *FCGR2C* haplotype in HIV-1 disease progression. Characterizing the mechanisms underlying the associations of c.134-96C>T in Thais and South Africans is imperative, particularly considering the genetic differences between the population groups. Moreover, establishing whether c.134-96C>T modifies the risk of HIV-1 acquisition in other models of persistent HIV-1 exposure, such as infants born to HIV-1-infected mothers and sero-discordant couples, may provide additional insight into its significance in the HVTN702 trial.

## Materials and methods

### Study participants

All study participants were HIV-1-infected black individuals recruited from hospitals and clinics in the city of Johannesburg, South Africa. HIV-1 controllers (*n* = 71) were recruited from hospitals in Soweto and Johannesburg. HIV-1 progressors (*n* = 73) were selected from the Soweto Lung Cohort (*n* = 756) recruited at Chris Hani Baragwaneth hospital. Ethical clearance was obtained from the University of the Witwatersrand Human Research Ethics Committee and all participants provided written informed consent.

### Genotyping

Genomic DNA from each participant was isolated from ethylenediaminetetraacetic acid (EDTA) whole blood obtained by venepuncture.*FCGR2C* copy number, expression variants c.169T>C (p.X57Q) and c.798+1A>G (rs76277413) and the promoter variant c.–386G>C (rs3219018) were genotyped using the *FCGR*-specific multiplex ligation-dependent probe amplification assay (MRC Holland, Amsterdam, The Netherlands) according to the manufacturer’s instructions. Amplicons were separated by capillary electrophoresis on an ABI Genetic Analyzer 3130 (Life Technologies, Applied Biosystems, Foster City, CA, USA) and fragments were analyzed with the Coffalyzer.NET software (MRC Holland) using peak height as a measure of gene/allele copy number.

The *FCGR2C* c.134-96C>T (rs114945036) and p.T118I (rs138747765) variants were genotyped through nucleotide sequencing. In brief, a 6374 base-pair fragment was amplified with the Expand Long Template PCR System (Roche, Mannheim, Germany) using the*FCGR2B/C* sense primer 5′-ATGTATGGGGTGTCTGTGTGTC-3′ and *FCGR2C*-specific antisense primer 5′-CTCAAATTGGGCAGCCTTCAC-3′ [14]. The PCR reaction consisted of ~20 ng of genomic DNA as template, 3.75 U Expand Long Template enzyme mix, 5 µl of 10 × PCR buffer 3 (2.75 mM MgCl_2_), 500 µM of each deoxynucleotide, 0.3 µM of each oligonucleotide primer and molecular-grade water to a final volume of 50 µl. The PCR conditions were 94 °C for 2 min, followed by ten cycles of 94 °C for 10 s, 60 °C for 15 s and 68 °C for 7 min, and 25 cycles of 94 °C for 15 s, 60 °C for 15 s and 68 °C for 7 min with an elongation of each subsequent cycle with 20 s, and a final elongation at 72 °C for 7 min. The internal antisense primer 5′-CCTCCACTGACCAGAAAGCAC-3′ was used in standard BigDye Terminator v3.1 Cycle Sequencing reactions. Sequences were analyzed in Sequencher version 4.5 (Gene Codes Corporation, Ann Arbor, MI, USA) and area under the curve of the electropherogram was used to determine allele count for individuals bearing more than two *FCGR2C* copies.

### Genetic variance description

Genetic variance description is according to the recommendations by the Human Genome Variation Society [24]. Gene, transcript and protein sequence accession numbers used to designate polymorphic variants are as follows: *FCGR2C*—NG_011982.1, NM_201563 and NP_963857.3. Variant coordinates are according to Ensembl human genome assembly GRCh38.p10.

### Statistical and computational analysis

Linkage disequilibrium was analyzed in Haploview and Arlequin v3.5 [25, 26]. Genotypic data for individuals with multiple gene copies were considered as homozygous if all copies carried the same allele or heterozygous when both alleles were detected. Data from the 1000 Genomes Project (phase 3) were used to assess linkage disequilibrium in other population groups. HaploReg v4.1 was used to identify potential effects of genetic variants on regulatory motifs and Human Splicing Finder v3.0 to predict alteration or creation of motifs involved in splicing [27].

The D’Agostino–Pearson omnibus normality test was used to determine the distribution of continuous variables. The *t* test was used to compare normally distributed continuous variables, the Mann–Whitney U test to compare non-normally distributed continuous variables, the Fisher’s exact test for categorical data and multivariate logistic regression to adjust for confounders. All statistical tests were two-sided. Analysis of genetic association between *FCGR2C* variants and HIV-1 control was restricted to variants with minor allele frequencies greater than 5% to reduce the number of tests and increase statistical power. Given the moderate sample size and the low frequency of minor allele homozygotes, the minor allele’s effect was tested under a dominant model of inheritance. We applied Bonferroni corrections to significant associations (indicated by *P*_Bonf_), which considered 12 independent tests—three unrelated clinical subgroups and four loci (CNR1, c.–386G>C, c.134-96C>T and c.169T>C [p.X57Q]). All analyses were performed in STATA version 10.1 (StataCorp LP, Texas, USA).
